# Gradient Profile Estimation Using Exponential Cubic Spline Smoothing in a Bayesian Framework

**DOI:** 10.3390/e23060674

**Published:** 2021-05-27

**Authors:** Kushani De Silva, Carlo Cafaro, Adom Giffin

**Affiliations:** 1 Department of Mathematics, Iowa State University, Ames, IA 50011, USA; 2 Department of Mathematics and Physics, SUNY Polytechnic Institute, Albany, NY 12203, USA; carlocafaro2000@yahoo.it; 3 Naval Nuclear Laboratory, Schenectady, NY 12309, USA; physics101@gmail.com

**Keywords:** computational techniques, inference methods, probability theory

## Abstract

Attaining reliable gradient profiles is of utmost relevance for many physical systems. In many situations, the estimation of the gradient is inaccurate due to noise. It is common practice to first estimate the underlying system and then compute the gradient profile by taking the subsequent analytic derivative of the estimated system. The underlying system is often estimated by fitting or smoothing the data using other techniques. Taking the subsequent analytic derivative of an estimated function can be ill-posed. This becomes worse as the noise in the system increases. As a result, the uncertainty generated in the gradient estimate increases. In this paper, a theoretical framework for a method to estimate the gradient profile of discrete noisy data is presented. The method was developed within a Bayesian framework. Comprehensive numerical experiments were conducted on synthetic data at different levels of noise. The accuracy of the proposed method was quantified. Our findings suggest that the proposed gradient profile estimation method outperforms the state-of-the-art methods.

## 1. Introduction

Estimating the gradient of a system from a discrete set of data has a vast number of applications in many fields, such as biology, engineering, and physics. For instance, determining the particle velocity from the discrete time-position data in particle image velocimetry and particle tracking velocimetry experiments is important in plasma physics [1,2,3,4]. Applications of velocity estimation in motion control systems using discrete-time data have increased with the invention of microprocessors (see [5], and the references therein). Moreover, with improved technology, faster equipment is now available to measure high-speed discrete data [1]. The state-of-the-art method for determining the gradient from data is to estimate an underlying smoothing function of the data and to take its subsequent analytic derivative [6,7,8,9,10,11,12,13] (similar approaches with Bayesian methods are used in [14,15,16,17,18,19]). Finite differencing is also used for this purpose [2]. However, when there is noise in the data, the results can be ill-posed because the derivative tends to blow up the uncertainty in the estimates [5]. The estimates become worse when these data are measured in shorter intervals, especially when position data are measured using high-speed cameras in particle tracking experiments [1,2].

On the other hand, the cubic splines used in these methods of estimating the data can produce drastic results depending on the recorded speed of data or noise levels. Exponential cubic splines are superior to cubic splines because of their capacity to capture abrupt changes in the data due to its extra parameter of smoothness [20,21]. However, special attention must be paid to this extra smoothing parameter because its extreme values can produce unrealistic results. In the literature, Jeffreys prior was used for this smoothing parameter as a means of scaling down [1,20,21].

In our study, motivated by the work in [1], we present a detailed investigation on gradient profile estimation as a Bayesian inference problem by directly estimating the gradient, which avoids taking the analytic derivative of noisy data. Moreover, we use exponential cubic spline as the underlying smoothing function of the gradient. We also introduce a more meaningful choice of prior distribution for the smoothing parameter of this spline. Moreover, we present a comprehensive sensitivity analysis of noise on the gradient estimates and, additionally, we present the estimates for position and acceleration obtained by the subsequent integration and derivative of our gradient estimates.

The paper is organized as follows. Section 2 develops the idea of separating spaces with mathematical background. The Bayesian framework of the algorithm is demonstrated in Section 3. Thereafter, the computational details are presented in Section 4 and Section 5. In particular, in these sections, our findings are compared to those obtained by means of more traditional methods, such as smoothing data with the subsequent analytic derivative. Our concluding remarks are given in Section 6.

## 2. Separating Spaces in Bayesian Context

In this section, we explain how the spaces are separated, which permits one to directly infer the gradient along with its mathematical forms. From this point forward, we use the terms (and mathematical notations) *x-space* (U) and *v-space* (V) to denote the space where measured data lives and the space where the gradient resides, respectively. We then use Bayes’ theorem to map the information between these two spaces (examples are given in [22,23,24,25,26,27,28]). The mapping is done using the obvious relationship between an object’s position and its velocity,

(1) $$x\left(t\right)\overset{\mathrm{def}}{=}\int v\left(t\right)dt,$$

where x(t) and v(t) represent the object’s position and velocity, respectively. By the use of our notations, x(t) lives in *x*-space and v(t) lives in *v*-space. In our approach, a functional form (mathematical model) is given to velocity, rather than to measured data as in the traditional approach. Therefore, our mapping is such that the positions in *x*-space are ’obtained’ by integrating the functional form placed in *v*-space. The Bayes’ theorem allows us to do this as an inference problem to infer the unknowns of the functional form in *v*-space with the information available in *x*-space.

The exponential cubic spline is proven to capture abrupt changes due to its high flexibility compared to the cubic spline [1,20,21]; thus we use the exponential cubic spline (denoted by $S_{v}$) as the functional form to represent the velocity, i.e., the exponential cubic spline lives in *v*-space (shown by the subscript *v*). This spline is the solution to the following minimal bending energy functional [1]:

(2) $$\int_{t_{1}}^{t_{n}}\left\{{\left[\frac{d^{2}S_{v}\left(t\right)}{dt^{2}}\right]}^{2}+\lambda {\left(t\right)}^{2}{\left[\frac{dS_{v}\left(t\right)}{dt}\right]}^{2}\right\}dt\;\mathrm{for}\;\xi_{v_{i}}\leq t\leq \xi_{v_{i+1}}.$$

for $S_{v}\left(\xi_{v_{i}}\right)=f_{v_{i}},i=1,\ldots ,E_{v}$ and $\lambda \left(t\right)=\lambda_{v_{i}}$ for $\xi_{i}\leq t\leq \xi_{i+1}$, where $t_{1}=\xi_{v_{1}}$ and $t_{n}=\xi_{v_{E_{v}}}$. Here, $f_{v_{i}}$ is the function value at $\xi_{v_{i}}$ and $\lambda_{v_{i}}$ is proportional to tension between two knots, $\xi_{v_{i}},\xi_{v_{i+1}}$. A numerically convenient reformulation of the spline at the *i*-th interval is then given by [21,29]

(3) $$\begin{array}{cc} \; & S_{v_{i}}\left(t,\;f_{v},\;\lambda_{v},\;\xi_{v},\;E_{v}\right)\overset{\mathrm{def}}{=}f_{v_{i}}\left(1-h_{v}\right)+f_{v_{i+1}}h_{v}\\ \; & +\frac{M_{v_{i}}}{\lambda_{v_{i}}^{2}}\left\{\frac{\sinh\left[\mu_{v_{i}}\left(1-h_{v}\right)\right]}{\sinh\left(\mu_{v_{i}}\right)}-\left(1-h_{v}\right)\right\}+\frac{M_{v_{i+1}}}{\lambda_{v_{i}}^{2}}\left[\frac{\sinh\left(\mu_{v_{i}}h_{v}\right)}{\sinh\left(\mu_{v_{i}}\right)}-h_{v}\right], \end{array}$$

for $i=1,\ldots ,E_{v}$. The quantities $h_{v}$, $\mu_{v_{i}}$, and $M_{v_{i}}$ in Equation (3) are defined as

(4) $$\begin{array}{c} h_{v}\overset{\mathrm{def}}{=}\frac{t-\xi_{v_{i}}}{\xi_{v_{i+1}}-\xi_{v_{i}}},\;\mu_{v_{i}}\overset{\mathrm{def}}{=}\lambda_{v_{i}}\left(\xi_{v_{i+1}}-\xi_{v_{i}}\right),\;\mathrm{and}\;\\ M_{v_{i}}\overset{\mathrm{def}}{=}{\left\{\frac{d^{2}}{dt^{2}}\left[S_{v_{i}}\left(t,\;f_{v},\;\lambda_{v},\;\xi_{v},\;E_{v}\right)\right]\right\}}_{t=\xi_{v_{i}}}, \end{array}$$

respectively. Using the arrow notations for vectorial quantities, the exponential cubic spline in Equation (3) can also be viewed in matrix form as [1]

(5) $$S_{v}\left(\vec{t}\right)=W\left(\vec{t},\;\vec{\lambda_{v}},\vec{\;\xi_{v}}\right)\vec{f_{v}}.$$

In Equation (5), *W* denotes the design matrix of $\vec{t}$-locations of the function values $\vec{f_{v}}$, the support points (knots) ${\vec{\xi }}_{v}$, and tension parameters ${\vec{\lambda }}_{v}$. The second derivative of $S_{v}$, $M_{v}$, can be found explicitly by solving the tri-diagonal system of equations in Equation (5).

We used the exponential cubic spline to model the gradient. Since the gradient is the unknown quantity here, the variables, $\vec{f_{v}},\;\vec{\lambda_{v}},\;\vec{\;\xi_{v}},\;E_{v}$ in Equation (3) are unknown. Therefore, it is important at this stage to identify data and parameters in this inference problem and they are given in Table 1.

Now, the general relationship given in Equation (1) can be rewritten precisely using to our model for an arbitrary *i*-th data point (i=1,…,n where *n* is the total number of measured data values) as

(6) $$x_{i}=\int_{t_{1}}^{t_{i}}S_{v}\left(t,\;f_{v},\;\lambda_{v},\;\xi_{v},\;E_{v}\right)dt,$$

with $S_{v}\left(t,\;f_{v},\;\lambda_{v},\;\xi_{v},\;E_{v}\right)$ given in Equation (3). That is, *i*-th position (*i*-th data point) is the integrated spline from first time point (time of the initial position) to the *i*-th time point (time of the *i*-th position). The relationship in Equation (6) can be further reduced to

(7) $$x_{i}=x_{i-1}+\int_{t_{i-1}}^{t_{i}}S_{v_{i-1}}\left(t,\;f_{v},\;\lambda_{v},\;\xi_{v},\;E_{v}\right)dt,\;2<i<n.$$

**Figure 1:**
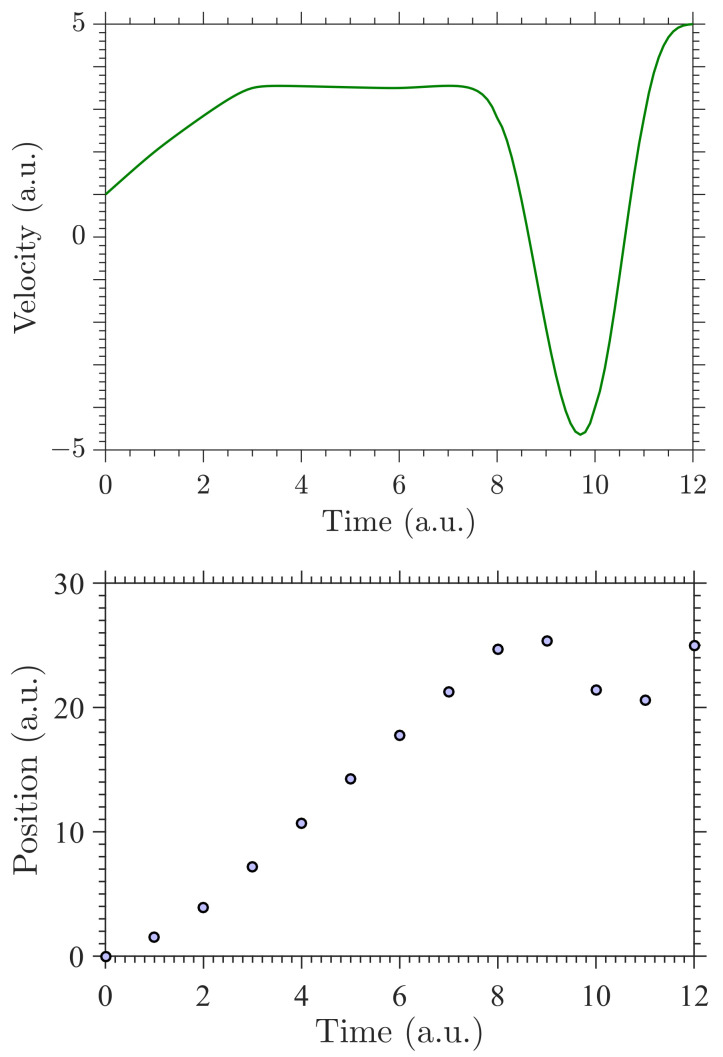
An example of solving a forward problem from a velocity curve (**top**) to calculate positions/distances (**bottom**). The velocity curve is obtained by the exponential cubic spline computed at $\vec{\xi }=\left[0,1,3,6,8,10,11,12\right]$. The positions were calculated at n=13 different time instances from the velocity by solving the forward problem. The time axis is given at arbitrary units (a.u.).

The starting and ending values of $x_{i}$ in Equation (7) are the initial and the last positions recorded. Here, $S_{v_{i-1}}$ is the spline in ${\left(i-1\right)}^{th}$ interval. We point out that the exponential cubic spline is analytically integrable and its technical details are given in Appendix A. When a relationship (as in (1) and (7)) is used to match an unknown quantity (gradient/velocity) and the observable data (position), the relationship should be able to generate data as close as possible to the observable information if the desired/unknown quantity is known. This is called the *forward problem* in Bayesian language. However, what is required here in our study is to be able to solve the *inverse problem*. That is, making inferences about the desired/unknown quantity using the observed information and the functional form of the unknown (here, the exponential cubic spline) [30,31,32]. An example of solving a forward problem by computing positions when the velocity is known is shown in Figure 1.

## 3. The Bayesian Recipe

The inverse problem for the position-velocity problem discussed in the previous section can be written using Bayes’ rule as follows:

(8) $$p\left(\mathrm{velocity}\in V|\mathrm{position}\in U\right)=\frac{p\left(\mathrm{velocity}\in V\right)p\left(\mathrm{position}\in U|\mathrm{velocity}\in V\right)}{p\left(\mathrm{position}\in U\right)},$$

which shows how the joint posterior probability distribution is built to infer the velocity (gradient) profile when the position profile is known. Bayes’ rule in Equation (8) can be rewritten using data and parameters given in Table 1 as follows:

(9) $$p\left(\vec{f_{v}},\;\vec{\lambda_{v}},\;\vec{\xi_{v}},\;E_{v}|\vec{t},\;\vec{x},\;I\right)=\frac{p\left(\vec{f_{v}},\;\vec{\lambda_{v}},\;\vec{\xi_{v}},\;E_{v}|I\right)p\left(\vec{x}|\vec{f_{v}},\;\vec{\lambda_{v}},\;\vec{\xi_{v}},\;E_{v},\;\vec{t},\;I\right)}{p\left(\vec{x}|I\right)},$$

where $p\left(\vec{x}|\vec{f_{v}},\;\vec{\lambda_{v}},\;\vec{\xi_{v}},\;E_{v},\;\vec{t},\;I\right)$ is the likelihood and the quantity *I* represents all the relevant background information. We assume the spline variables are independent. It then enables us to write the following:

(10) $$p\left(\vec{f_{v}},\;\vec{\lambda_{v}},\;\vec{\xi_{v}},\;E_{v}|I\right)=p\left(\vec{f_{v}}|I\right)p\left(\vec{\lambda_{v}}|I\right)p\left(\vec{\xi_{v}}|I\right)p\left(E_{v}|I\right).$$

The special case of when the number of knots and the position of knots are known (decided prior to computing the spline), we can write  (10)

(11) $$p\left(\vec{f_{v}},\;\vec{\lambda_{v}},\;\vec{\xi_{v}},\;E_{v}|I\right)=p\left(\vec{f_{v}}|E_{v},\;\vec{\xi_{v}},\;I\right)p\left(\vec{\lambda_{v}}|E_{v},\;\vec{\xi_{v}},\;I\right).$$

Substituting Equation (10) into Equation (9), we get the general form of the posterior distribution:

(12) $$\begin{array}{c} p\left(\vec{f_{v}},\;\vec{\lambda_{v}},\;\vec{\xi_{v}},\;E_{v}|\vec{t},\;\vec{x},\;I\right)=\frac{p\left(\vec{f_{v}}|I\right)p\left(\vec{\lambda_{v}}|I\right)p\left(\vec{\xi_{v}}|I\right)p\left(E_{v}|I\right)p\left(\vec{x}|\vec{f_{v}},\;\vec{\lambda_{v}},\;\vec{\xi_{v}},\;E_{v},\;\vec{t},\;I\right)}{p(\vec{x}|I)}. \end{array}$$

The evidence $p\left(\vec{x}|I\right)$ in Equation (9) is given by

$$\begin{array}{c} p\left(\vec{x}|I\right)\overset{\mathrm{def}}{=}\;\int \int \int \int \;p\left(\vec{f_{v}}|I\right)p\left(\vec{\lambda_{v}}|I\right)p\left(\vec{\xi_{v}}|I\right)p\left(E_{v}|I\right)p\left(\vec{x}|\vec{f_{v}},\;\vec{\lambda_{v}},\;\vec{\xi_{v}},\;E_{v},\;\vec{t},\;I\right)d\vec{f_{v}}d\vec{\lambda_{v}}d\vec{\xi_{v}}dE_{v}. \end{array}$$

The relationship between the gradient and position in Equation (7) is included in the likelihood. We then assume the noise (or the uncertainty) in the measured data, $\vec{\epsilon }\overset{\mathrm{def}}{=}\left(\epsilon_{1},\ldots ,\epsilon_{n}\right)$, has a Gaussian process with

(13) $$\vec{\epsilon }∼N\left(\vec{\mu },\;\Sigma \right),$$

where the quantity $\vec{\mu }$ is the $\left(n\times 1\right)$-dimensional mean vector and Σ is a $\left(n\times n\right)$-dimensional covariance matrix of the noise. We further assume $\vec{\mu }=0$ and the noise is uncorrelated which, in turn, makes Σ a diagonal matrix with diagonal elements, $\left\{\sigma_{1},\ldots ,\;\sigma_{n}\right\}$. Omitting the arrow notation for vectorial quantities, we can write the following:

(14) $$\begin{array}{c} x_{i}=\left\{\begin{array}{cc} x_{1} & +\sum_{\gamma =1}^{i-1}\left(\int_{\xi_{v_{\gamma }}}^{\xi_{v_{\gamma +1}}}S_{v_{\gamma }}\left(t,\;f_{v},\;\lambda_{v},\;\xi_{v},\;E_{v}\right)dt\right)\\ \; & +\int_{\xi_{v_{i}}}^{t_{i}}S_{v_{i}}\left(t,\;f_{v},\;\lambda_{v},\;\xi_{v},\;E_{v}\right)dt+\epsilon_{i},\;\mathrm{for}\xi_{v_{i}}<t_{i}<\xi_{v_{i+1}}\\ x_{1} & +\sum_{\gamma =1}^{i-1}\left(\int_{\xi_{v_{\gamma }}}^{\xi_{v_{\gamma }+1}}S_{v_{\gamma }}\left(t,\;f_{v},\;\lambda_{v},\;\xi_{v},\;E_{v}\right)dt\right)+\epsilon_{i},\;\mathrm{for}t_{i}=\xi_{v_{i}} \end{array}\right. \end{array}$$

In Equation (14), the first line (case 1) showcases when the time $t_{i}$, in which $x_{i}$ is measured, falls between two knots of the spline, in which case $S_{v_{i}}$, calculated from $\xi_{v_{i}}$ to $t_{i}$, is a partial interval. The second line (case 2) showcases when $t_{i}$ falls exactly on a knot, in which case $S_{v_{\gamma }}$, calculated at $t_{i}=\xi_{v_{i}}$, is a full interval. Note that case 2 is a special case of case 1. In other words, case 2 can be achieved via case 1 when $t_{i}=\xi_{v_{i}}$. Therefore, we rewrite Equation (14) for the general case subjecting the noise:

(15) $$\epsilon_{i}=x_{i}-\left(x_{1}+\sum_{\gamma =1}^{i-1}\int_{\xi_{v_{\gamma }}}^{\xi_{v_{\gamma +1}}}S_{v_{\gamma }}\left(t,\;f_{v},\;\lambda_{v},\;\xi_{v},\;E_{v}\right)dt+\int_{\xi_{v_{i}}}^{t_{i}}S_{v_{i}}\left(t,\;f_{v},\;\lambda_{v},\;\xi_{v},\;E_{v}\right)dt\right),\;$$

for i=1,…,n. By the assumption of iid (independent and identically distributed), we write our likelihood function:

(16) $$p\left(\vec{x}|\vec{f_{v}},\;\vec{\lambda_{v}},\;\vec{\xi_{v}},\;E_{v},\;\vec{t},\;I\right)=\prod_{i=1}^{n}p\left(x_{i}|\vec{f_{v}},\;\vec{\lambda_{v}},\;\vec{\xi_{v}},\;E_{v},\;t_{i},\;I\right).$$

Combining Equations (15) and (16) together with the additional assumption that $\sigma_{i}=\sigma_{e}=$ constant for any 1≤i≤n, the likelihood distribution becomes (case 1 of Equation (14) is assumed here for generality)

(17) $$\begin{array}{cc} p(\vec{x}|\vec{f_{v}},\;\vec{\lambda_{v}},\;\vec{\xi_{v}},\;E_{v},\;\vec{t},\;I) & =\prod_{i=1}^{n}\frac{1}{\sqrt{2\pi \sigma_{e}^{2}}}\exp\left[-\frac{1}{2\sigma_{e}^{2}}{\left(\epsilon_{i}\right)}^{2}\right]\\ \; & ={\left(2\pi \sigma_{e}^{2}\right)}^{-n/2}\exp\sum_{i=1}^{n}G\left(x_{i},\;\vec{f_{v}},\;\vec{\lambda_{v}},\;\vec{\xi_{v}},\;E_{v},\;\vec{t}\right), \end{array}$$

where,

(18) $$G\left(x_{i},\;\vec{f_{v}},\;\vec{\lambda_{v}},\;\vec{\xi_{v}},\;E_{v},\;\vec{t}\right)\overset{\mathrm{def}}{=}-\frac{1}{2\sigma_{e}^{2}}{\left[x_{i}-\left(x_{1}+\sum_{\gamma =1}^{i-1}\int_{\xi_{v_{\gamma }}}^{\xi_{v_{\gamma +1}}}S_{v_{\gamma }}\left(\cdot \right)dt+\int_{\xi_{v_{i}}}^{t_{i}}S_{v_{i}}\left(\cdot \right)dt\right)\right]}^{2}.$$

### The Choice of Prior Probability Distributions

According to Equation (11), we have three prior distributions to look at. We assume that there is no prior information about the spline values, $p\left(\vec{f_{v}}|E_{v},\;I\right)$, or the position of knots, $p\left(\vec{\xi_{v}}|E_{v},\;I\right)$. However, we give our attention to the tension parameter, ${\vec{\lambda }}_{v}$, because the shape of the spline between knots depends on it. The tension value is a scale parameter and it can take any real number. With the change in $\lambda_{v_{i}}$ from 0 to *∞*, the spline changes from a polygonal function to a cubic spline [21,29]. A polygonal function lacks the smoothness required by a moving object and the cubic spline can sometimes overestimate the curvature by producing cubic curves when knots are placed close. Hence, estimating an optimum tension value plays a big role in getting a reliable gradient estimate profile and choosing an appropriate prior adds a great value to it.

Previous studies in the literature [21,33] used Jeffreys prior to merely scale down large tension values to avoid unrealistic results. In this work, we used a Gaussian distribution as a prior for the tension (Jeffreys and uniform priors were used in previous work by the same authors related to this problem [34]) because: (1) it allows one to have a higher probability for sensible values for the spline and lower probability for nonsensible values for the spline; and (2) a conjugate prior makes the posterior a Gaussian family of distribution (the known parametric form), thus making the computations easy. As stated earlier, polygonal functions lack the smoothness of a moving object and thus values of tension that produce polygonal functions are considered nonsensible values in this problem.

Assuming the prior distribution of tension parameters, $\vec{\lambda_{v}}$, are iid Gaussian distributions with mean $\mu_{i}$ and variance $\sigma_{\theta_{\lambda_{i}}}$, we can write

(19) $$\begin{array}{cc} p(\vec{\lambda_{v}}|E_{v},\;I) & =\prod_{i=1}^{E_{v}-1}p\left(\lambda_{v_{i}}|E_{v},\;I\right)\\ \; & =\prod_{i=1}^{E_{v}-1}\frac{1}{\sqrt{2\pi \sigma_{\theta_{\lambda_{i}}}^{2}}}\exp\left[-\frac{{\left(\lambda_{v_{i}}-\mu_{i}\right)}^{2}}{2\sigma_{\theta_{\lambda_{i}}}^{2}}\right]\\ \; & ={\left(2\pi \sigma_{\theta_{\lambda }}^{2}\right)}^{-(E_{v}-1)/2}\exp\left[-\sum_{i=1}^{E_{v}-1}\frac{{\left(\lambda_{v_{i}}-\mu_{i}\right)}^{2}}{2\sigma_{\theta_{\lambda }}^{2}}\right]\\ \; & (\mathrm{by}\;\mathrm{taking}\;\sigma_{\theta_{\lambda_{i}}}=\sigma_{\theta_{\lambda }}\forall i\;\mathrm{with}\;1\leq i\leq E_{v}-1). \end{array}$$

Finally, the posterior distribution $p\left(\vec{f_{v}},\;\vec{\lambda_{v}},\;\vec{\xi },\;E_{v}|\vec{t},\;\vec{x},\;I\right)$ in Equation (9) can be written as

$$\begin{array}{cc} \; & p\left(\vec{f_{v}},\;\vec{\lambda_{v}},\;\vec{\xi },\;E_{v}|\vec{t},\;\vec{x},\;I\right)\propto p\left(\vec{\lambda_{v}}|E_{v},\;I\right)p\left(\vec{x}|\vec{f_{v}},\;\vec{\lambda_{v}},\;\vec{\xi },\;E_{v},\;\vec{t},\;I\right)\\ \; & \propto {\left(2\pi \sigma_{\theta_{\lambda }}^{2}\right)}^{-(E_{v}-1)/2}{\left(2\pi \sigma_{e}^{2}\right)}^{\left(-n/2\right)}\exp\left[-\sum_{i=1}^{E_{v}-1}\frac{{\left(\lambda_{v_{i}}-\mu_{i}\right)}^{2}}{2\sigma_{\theta_{\lambda }}^{2}}+\sum_{i=1}^{n}G\left(x_{i},\;\vec{f_{v}},\;\vec{\lambda_{v}},\;\vec{\xi },\;E_{v},\;\vec{t}\right)\right], \end{array}$$

where $G(x_{i},\;\vec{\vec{f_{v}}},\;\vec{\lambda_{v}},\;\vec{\xi_{v}},\;E_{v},\;\vec{t})$ is given in Equation (18). In this work, the Gaussian prior distribution uses $\mu_{i}=0$ and $\sigma_{\theta_{\lambda }}=5$. As noted previously [34], even though lambda has a range of 0 to *∞*, the impact of lambda on the spline starts to flatten around 10. Thus, by setting $\sigma_{\theta_{\lambda }}=5$, $2\sigma_{\theta_{\lambda }}$ covers 95% of the prior distribution.

## 4. Numerical Simulation

In this section, we discuss the numerical method followed to simulate the posterior distribution for inferring the velocity/gradient using positions/distances. We first created synthetic data from the velocity curve showcased for forward problem in Figure 1. We generated a sample of n=121 position data points. Thereafter, we added Gaussian noise of μ=0 and different levels of $\sigma_{e}$ to create different datasets of different noise levels ($\sigma_{e}$). In this work, we hand-picked the number of knots and their positions for simplicity and to demonstrate the inference (Equation (11)). Thus, we have $2E_{v}-1$ number of parameters to infer: spline values, $f_{v}$, and tension values $\lambda_{v}$. However, in future work, it is important that these two sets of parameters are inferred.

For the synthetic data, following the work in [29], we selected $E_{v}=8$ knots at $\vec{\xi_{v}}=\left[0,1,3,6,8,10,11,12\right]$ in inferring the spline. Then, the posterior distribution with different priors (see Equation (12)) were simulated using a hybrid MCMC algorithm called DRAM [35,36]. The simulation begins with an initial minimization process. It should be noted that the posterior distribution is undefined at $\lambda_{v}=0$ due to numerical instability of the spline. Therefore, the smallest value that was used for $\lambda_{v}$ was 0.001. Thus, the values of the parameters were initialized with the following values for the initial minimization process:

(20) $$f_{v_{\mathrm{initial}}}=0,\;\mathrm{and}\;\lambda_{v_{\mathrm{initial}}}=0.001.$$

The initial minimization acts as a jump start to the DRAM algorithm. The proposal distributions of the parameters used in DRAM that generate the candidate sample values are Gaussian distributions. The parameter values of the proposal distributions (i.e., the mean and variance of Gaussian distributions) are user inputs and are showcased in Table 2, where LB and UB are the lower and upper bounds of the parameters, respectively.

The optimum values ${\vec{f_{v}}}^{*}$ and ${\vec{\;\lambda_{v}}}^{*}$ produced by the initial minimization were used as the mean values of the proposal distributions ($\mu_{v_{i}},\;\mu_{\lambda_{i}}$), so that the initial proposal distributions are centered around ${\vec{f_{v}}}^{*}$ and ${\vec{\;\lambda_{v}}}^{*}$. The information of prior distributions can be specified with prior means $\mu_{\theta_{v_{i}}}$, $\mu_{\theta_{\lambda_{i}}}$ and prior variances $\sigma_{\theta_{v_{i}}},\;\sigma_{\theta_{\lambda_{i}}}$. The DRAM procedure takes the variance of the prior as the initial variance of the parameters if the covariance matrix is not specified by the user. When the prior variance is not specified, the variances of the proposal distributions are calculated as

(21) $$\sigma_{v_{i}}^{2}={\left(\mu_{v_{i}}\times 0.05\right)}^{2},\;\mathrm{and}\;\sigma_{\lambda_{i}}^{2}={\left(\mu_{\lambda_{i}}\times 0.05\right)}^{2}.$$

In other words, if no prior information is available about the variance of the parameter, 5% of the mean of the initial distribution is taken as the initial variance. Finally, samples were drawn from these Gaussian proposals and the marginal densities of the parameters were computed. The MCMC simulations were run until convergence was observed. Each sampling procedure of length Ω was repeated M=100 times. At each of the M-th repetitions, a different dataset of same noise level was created followed by an initial minimization process. All the sample chains were stored and, in addition, for a given sample chain, the first few samples were burnt during Ω×p% period. The quantity *p* denotes the burn-in time. The average of M marginal densities of each parameter were then computed. In what follows, we assume that ω denotes the remaining number of samples after the burn-in period, that is, $\omega \overset{\mathrm{def}}{=}\left(1-p\right)\%\times \Omega$. Then, for any $k=1,\ldots ,E_{v}$, the mean values $\left\{f_{v_{k}}^{*}\right\}$ of those average marginal densities were calculated as

(22) $$f_{v_{k}}^{*}\overset{\mathrm{def}}{=}\frac{1}{\omega }\sum_{i=1}^{\omega }{\bar{f_{v}}}_{ki},$$

where ${\bar{\;f_{v}}}_{ki}$ denote the average sample values of $f_{v_{k}}$ after M number of iterations,

(23) $${\bar{f_{v}}}_{ki}\overset{\mathrm{def}}{=}\frac{1}{M}\sum_{j=1}^{M}f_{v_{kij}}.$$

Following the same line of reasoning presented for Equations (22) and (23), we also have

(24) $$\lambda_{v_{s}}^{*}\overset{\mathrm{def}}{=}\frac{1}{\omega }\sum_{i=1}^{\omega }{\bar{\lambda_{v}}}_{si}=\frac{1}{\omega M}\sum_{i=1}^{\omega }\sum_{j=1}^{M}\lambda_{v_{sij}},$$

for any $s=1,\ldots ,E_{v}-1$ with ${\bar{\lambda_{v}}}_{si}$ denoting the average sample values of tension after M number of iterations:

(25) $${\bar{\lambda_{v}}}_{si}\overset{\mathrm{def}}{=}\frac{1}{M}\sum_{j=1}^{M}\lambda_{v_{sij}}.$$

Finally, the standard deviations $\left\{\sigma_{v_{k}}^{*}\right\}$ and $\left\{\sigma_{\lambda_{s}}^{*}\right\}$ of the parameters are calculated from the average of all chains after M iterations as

(26) $$\sigma_{v_{k}}^{*}\overset{\mathrm{def}}{=}\sqrt{\frac{1}{\omega -1}\sum_{i=1}^{\omega }{\left({\bar{f_{v}}}_{ki}-f_{v_{k}}^{*}\right)}^{2}},$$

for any $k=1,\ldots ,E_{v}$ and

(27) $$\sigma_{\lambda_{s}}^{*}\overset{\mathrm{def}}{=}\sqrt{\frac{1}{\omega -1}\sum_{i=1}^{\omega }{\left({\bar{\lambda }}_{v_{si}}-\lambda_{v_{s}}^{*}\right)}^{2}},$$

for any $s=1,\ldots ,E_{v}-1$, respectively.

## 5. Results

**Figure 2:**
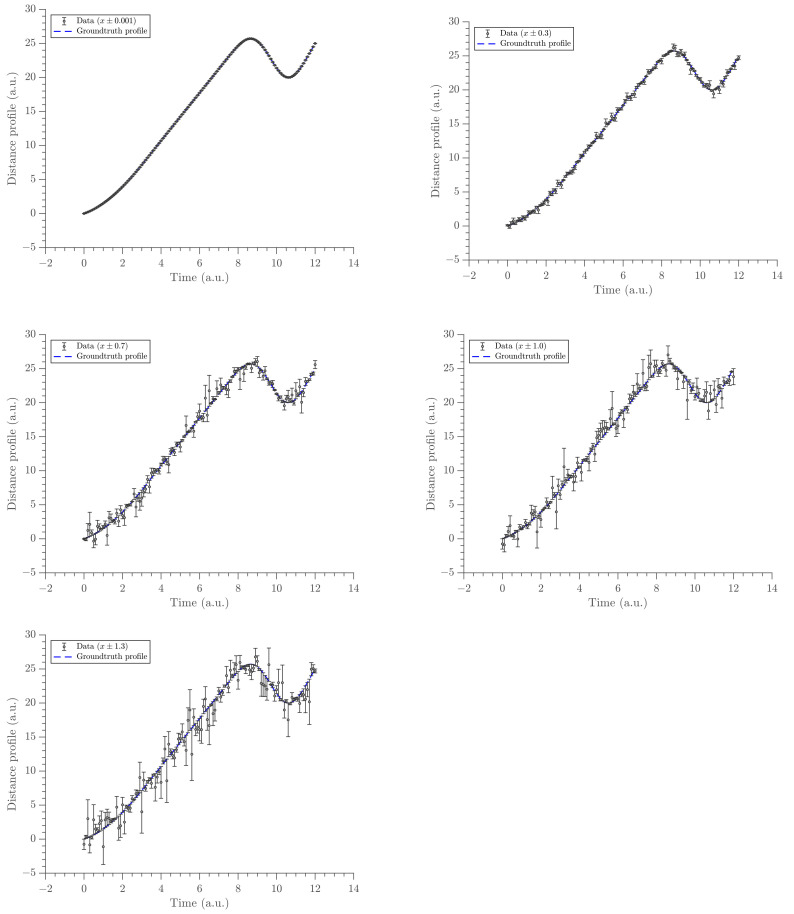
The panel of time-position data with error bars at five different noise levels. Noise levels increase from left to right and top to bottom. The length of error bars is relative to the magnitude of the noise.

This section presents the results obtained from the methodology built throughout the previous sections. The sensitivity of gradient estimates at different noise levels (of the position data) were tested. The noise levels (denoted by $\sigma_{e}$) on *x*-space are depicted in Figure 2 with five different noise levels: $\sigma_{e}\in \;\left\{0.001,\;0.3,\;0.7,\;1.0,\;1.3\right\}$. The noise levels were selected so that they cover almost all possibilities of the standard Gaussian noise. That is, by the definition of the Gaussian distribution, the first four noise levels cover a probability of 68.2% and the last noise level covers a probability of 95%.

The sensitivity of noise levels to the posterior distribution was investigated when the Gaussian prior was used for the tension parameter. These results of the sensitivity analysis were compared against a common traditional method of fitting the same type of exponential cubic spline in *x*-space and the gradient was obtained by analytically differentiating the resulting fitted spline function. The notation “*i*-fit (*i*-space) with i= x, v” denotes that *i*-fit was fitted on the *i*-space, where the x-fit is the distance profile, while the v-fit is the gradient profile.

**Figure 3:**
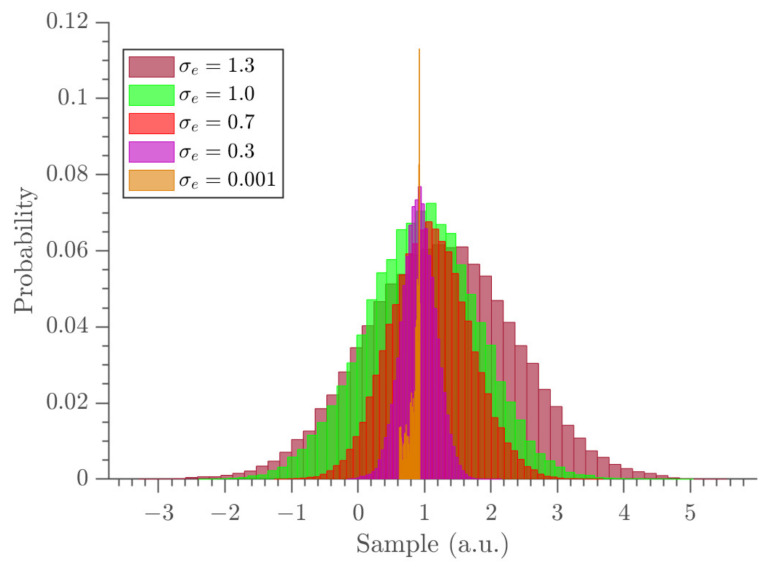
The comparison of marginal probability densities of the $f_{v_{1}}$ parameter at all noise levels.

**Figure 4:**
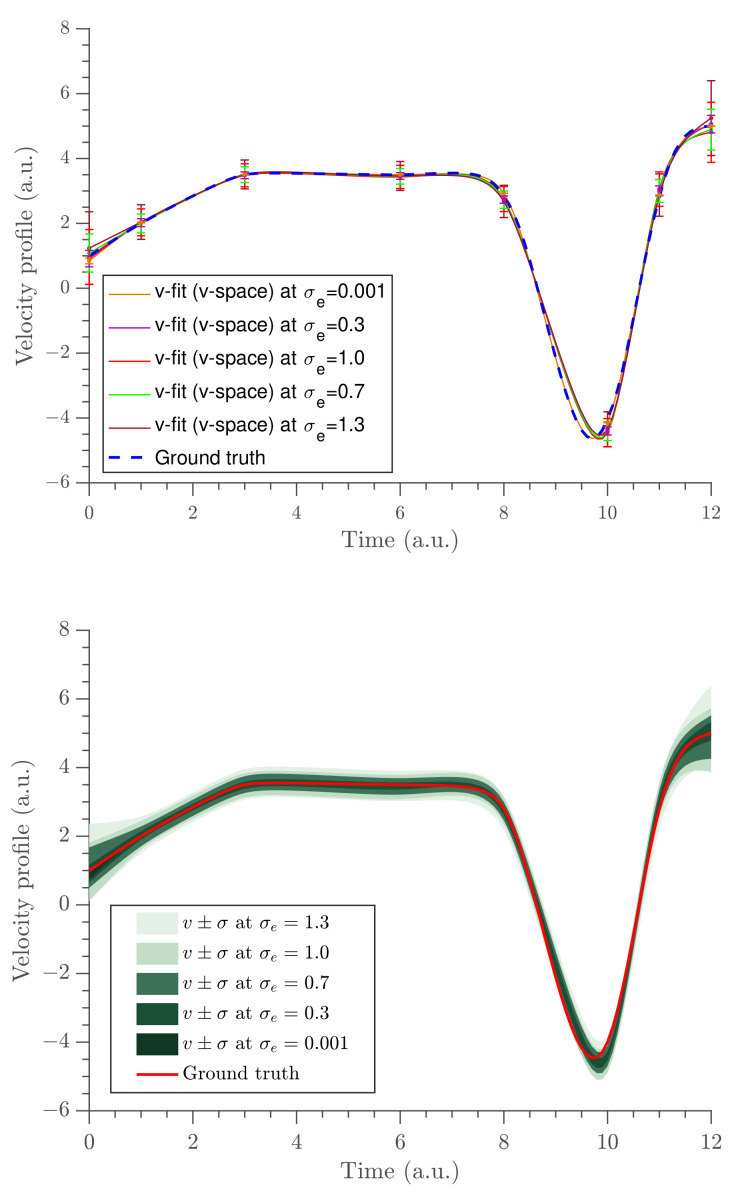
The gradient estimates obtained at the five different noise levels. The uncertainty of the velocity estimates (${\hat{\sigma }}_{v}$) are shown using error bars (**top** panel) and credible intervals (**bottom** panel).

A constant force was observed between the times 0–8 (a.u.) and short impulses were observed between the times 8–11 (a.u.). Moreover, it was observed that as the noise level increased, the data around the short impulses tended to become fuzzier so that it was hard to identify the trajectory. When the noise increased, the width of the marginal distributions were expanded. This, in turn, resulted in an increased length of the error bars of the parameter estimates. As a demonstration, we show the marginal probability distribution of $f_{v_{1}}$ in Figure 3 at all noise levels. The gradient estimates at all noise levels are depicted in Figure 4 and the uncertainties of $f_{v_{i}}$ parameters are shown in the same figure using error bars (top panel) and Bayesian credible intervals (bottom panel). The estimates almost overlap with the ground truth data at all noise levels, except at the boundaries and near t=10 (a.u.), where the short impulses were present in the position profile.

**Figure 5:**
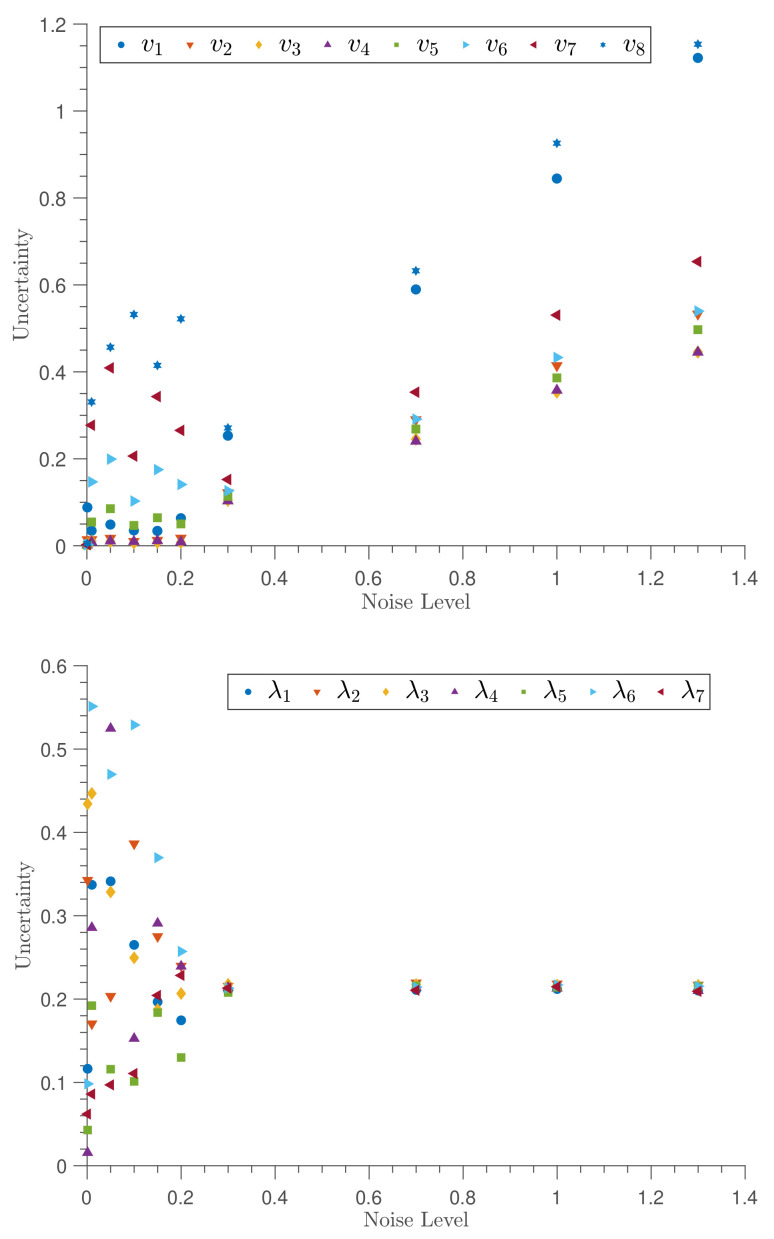
The relationship between uncertainty of the estimates and the noise level of the system. The top panel depicts the relationship of velocity parameters (here, $v_{i}$ indicates $f_{v_{i}}$), whereas the bottom panel shows the relationship of tension parameters (here, $\lambda_{i}$ indicates $\lambda_{v_{i}}$ ). Note that additional noise levels are included to demonstrate how the uncertainty in the estimated lambda converges to the Gaussian prior uncertainty when the data noise exceeds the prior uncertainty.

The uncertainties of both velocity and tension parameters are characterized in Figure 5. The uncertainties of the velocity parameters with the noise level exhibit a linear relationship reflecting the linear relationship of velocity parameters in the likelihood function. The uncertainties of the tension parameters converge to the Gaussian prior uncertainty when the data noise exceeds the prior uncertainty. To help illustrate this, additional noise levels were added in these graphs in Figure 5 to show the relationship more clearly.

**Figure 6:**
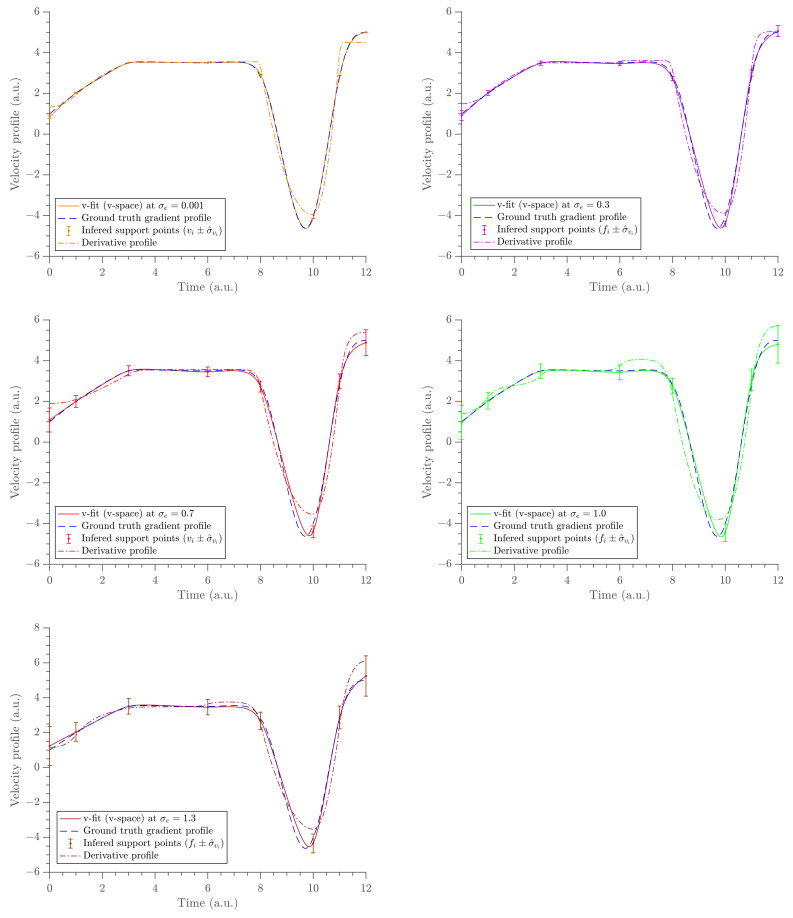
The panel of velocity estimates from the two spaces compared at five different noise levels. The noise level increases from left to right and top to bottom. v-fit (*v*-space) shows the gradient inferred by placing the exponential cubic spline in *v*-space (solid lines). The derivative profile shows the gradient estimates obtained by differentiating the exponential cubic spline fit in *x*-space (dashed-dot lines).

**Figure 7:**
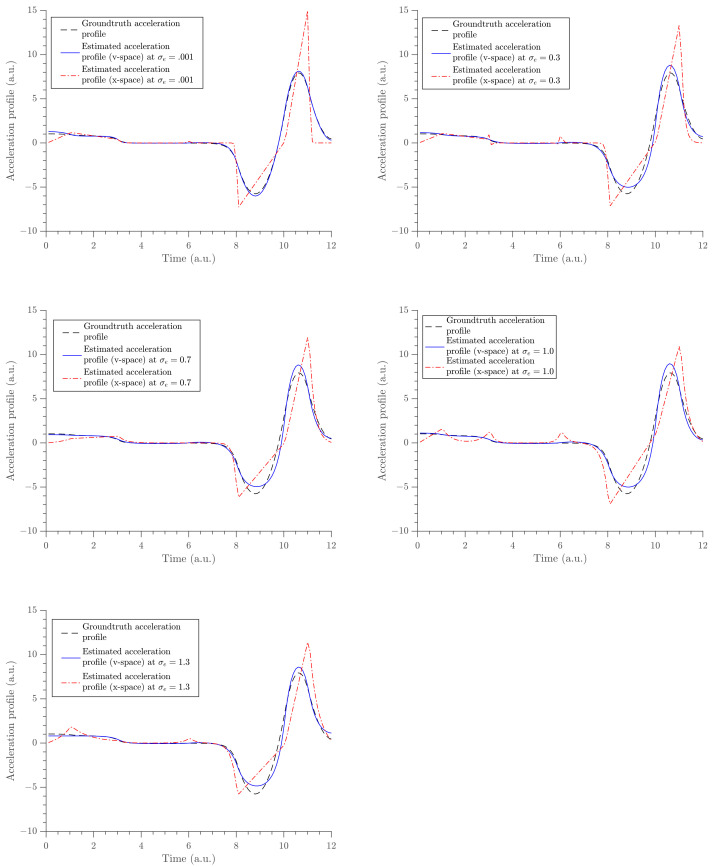
The panel of acceleration estimates obtained from the two methods are compared at five different noise levels. Solid blue lines shows the estimates obtained from our method and dashed-dot red lines shows the estimates obtained from the traditional method.

**Figure 8:**
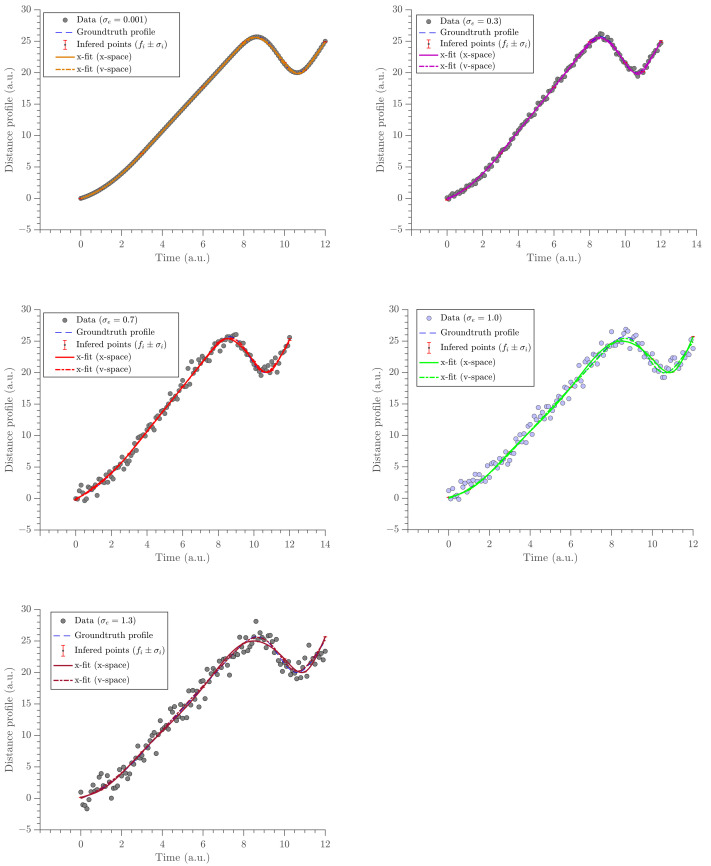
The panel of x-fit estimates obtained from two different methods compared at five different noise levels. Dashed-dot lines shows the estimates obtained from our method and solid lines shows the estimates obtained from the traditional method.

The gradient estimates are compared with the analytic derivative of the x-fit obtained in U. The x-fit is obtained by fitting the same exponential cubic spline to the time-position data in U. This was performed at the same knot positions. The gradient profile, the acceleration profile, and the position profile were obtained from the two methods (via v-fit from V and x-fit from U) and are compared in Figure 6, Figure 7 and Figure 8, respectively. The acceleration profile from our method (v-fit in V) was obtained by differentiating the exponential cubic spline, i.e., the analytic derivative of exponential cubic spline. It should be noted that ideally the acceleration should be inferred directly. Then, velocity and position could be found via integration. However, the focus and motivation of this paper was velocity. We are only using this as a quick comparison to illustrate how poor the acceleration fit would be when differentiating twice. The same was obtained using the traditional method by finite-differencing its gradient profile, i.e., finite differencing the analytic derivative of the exponential cubic spline.

The gradient estimates that emerge from the two spaces (namely, the *x*-space and *v*-space) clearly show some remarkable differences. In particular, the difference in accuracy is shown in Table 3 using the error norm:

(28) $${\left||e|\right|}^{2}\overset{\mathrm{def}}{=}\sum_{i=1}^{L}{\left(f_{v_{{\mathrm{estimated}}_{i}}}-f_{v_{{\mathrm{true}}_{i}}}\right)}^{2},$$

where *L* is the sample size [37].

The acceleration characterizes the acting forces on the object. The acceleration estimate (*x*-space) could not achieve constant force where it is expected. The errors at short impulses around t=10 a.u. started becoming very large, which indicates the possibility of infinite forces. Moreover, the acceleration profile from the *x*-space (red dashed-dot lines) is not realistic with its very sharp turns. Therefore, the acceleration estimate from the velocity space is a reliable estimator of acting forces. The goodness of fit was tested by studying the squared sum of errors (SSE) defined as [38]

(29) $$\mathrm{SSE}\overset{\mathrm{def}}{=}\sum_{i=1}^{n}{\left({\hat{f}}_{i}-f_{i}\right)}^{2}$$

where ${\hat{f}}_{i}$ represent the estimated function values. The quantity $f_{i}$ in Equation (29) is replaced with noisy $f_{i}$ to obtain the second and the third columns in Table 4, while $f_{i}$ is replaced with the original $f_{i}$ without noise to calculate the fourth and fifth columns in the same table.

The trajectory estimate (position profiles) is a bonus product from the gradient profile (*v*-space) and is obtained by integrating the gradient estimate (*v*-space). They are compared in Figure 8. The x-fit (*x*-space) follows the noisy data closely. This is because, when fitting a curve in the same space as data, that curve is trying to minimize the SSE, which means it tries to match the data as much as possible. Therefore, the x-fit from U tries to satisfy the noisy data as much as possible. However, the x-fit (*v*-space) follows the ground truth data more than it follows the noisy data (see Table 4). When integrating the v-fit (*v*-space), an additional order of differentiability is added to the trajectory estimate. Therefore, the x-fit (*v*-space) has a higher order of differentiability than that of the x-fit (*x*-space). This additional order of differentiability ensures the continuity of the velocity and, therefore, it satisfies the constraint of the finite force of the object.

The calculated SSE with true data and noisy data are shown in the Table 4. The SSE values for noisy data are smaller for the x-fit (*x*-space). This, in turn, reflects the fact that the x-fit (*x*-space) better follows noisy data. However, the SSE values for true data are smaller for the x-fit (*v*-space). This, instead, reflects the fact that the x-fit (*v*-space) better follows true data.

## 6. Conclusions

In this paper, we proposed a method to compute the gradient profile of a noisy system using the Bayesian inference method. It allowed us to infer the unobservable quantity, the gradient (velocity), by building a meaningful relationship between the unobservable quantity in velocity space and the observable quantity in data space. Furthermore, the unobservable quantity (the gradient) was modeled using the exponential cubic spline in velocity space. Using Bayesian methods, the parameters of this exponential cubic spline were inferred. The results of this new method were compared against those of a traditional method of modeling noisy data, which uses the exponential cubic spline in data space and takes the subsequent analytic derivative to obtain the gradient.

The results show that the gradient estimates obtained by modeling in velocity space are more accurate than the estimates obtained via the traditional method (see Table 3). Moreover, our method was able to produce better acceleration estimates with reliable and accurate values, where a constant force is expected (see Figure 7). We also compared the trajectory profile. We conclude that when the traditional method is used, the trajectory estimates tend to follow noisy data, whereas when our method is used, the estimates tend to follow the ground truth data, which, in return, produce more accurate estimates (see Figure 8 and Table 4). It is argued that by integrating the model in velocity space to compute the trajectory values, an extra order of differentiability is added. This argument can be further extended to suggest that the acceleration should be inferred first. Then, velocity and trajectories can be inferred by integration. However, this was not the focus of the paper. In conclusion, the method demonstrated in this paper is superior when estimating the velocity of a moving object under finite force as compared to others in the literature (for instance, see [1] and the references therein). It provided better results in all three estimates: trajectory, velocity, and acceleration.

It is necessary to note that, although our main focus in this paper was on the proposal of a novel theoretical method to estimate a gradient profile from discrete noisy data, a number of improvements can be sought in its practical implementation. For instance, the use of an MCMC algorithm gains precision at the expense of speed. A faster algorithm (such as the expectation maximization algorithm [39]) may be needed for real-time estimation. Furthermore, the amount and placement of the knots lacks a systematic guiding principle. In the future, we plan to use Bayesian model selection for determining the amount of knots. For the placement issue, we can adopt a hierarchical approach by including spline knot location algorithms [40] in conjunction with our main algorithm. This would provide estimates for the values associated with the knots and where they should be located. Finally, we hope to apply our Bayesian estimation technique to more realistic problems in which acceleration, velocity, and trajectory estimations are needed. In particular, from both theoretical and applied perspectives, in the future, we think it may be worth exploring the possibility of characterizing velocity profiles in ferromagnetic fluids [41] with the use of entropic inference methods that encompass Bayesian techniques [42] and, moreover, show promising results in inferring the ferromagnetic properties of materials [43].

## Figures and Tables

**Table 1:** The definition of data and parameters of the inference problem. The spaces where they reside are given in parentheses.

Data (*x*-space)	$\;\;\;\;\;\vec{t},\;\vec{x}$
Parameters (*v*-space)	${\vec{f}}_{v}$, ${\vec{\lambda }}_{v}$, ${\vec{\xi }}_{v},\;E_{v}$

**Table 2:** The sets of parameters in initial probability distributions of DRAM.

Parameter	Proposal Distribution	Prior Distribution
$f_{v_{i}}$ for $i=1,\ldots ,E_{v}$	Gaussian ($\mu_{v_{i}},\;\sigma_{v_{i}}^{2}$) $\in (LB_{v_{i}},\;UB_{v_{i}})$	$\mu_{\theta_{v_{i}}},\;\sigma_{\theta_{v_{i}}}$
$\lambda_{v_{i}}$ for $i=1,\ldots ,E_{v}-1$	Gaussian ($\mu_{\lambda_{i}},\;\sigma_{\lambda_{i}}^{2}$) $\in (LB_{\lambda_{i}},\;UB_{\lambda_{i}})$	$\mu_{\theta_{\lambda_{i}}},\;\sigma_{\theta_{\lambda_{i}}}$

**Table 3:** The comparison of accuracy of velocity fit from our method (in v-fit (*v*-space)) and the traditional method (derivative of x-fit in *x*-space).

	${\left||e|\right|}^{2}$	
**Noise Level**	**v-Fit (v-Space)**	**Derivative** **of x-Fit (x-Space)**
0.001	0.2077	14.4107
0.3	1.9679	15.0792
0.7	3.1793	20.9974
1	3.1569	30.1122
1.3	3.8159	31.2719

**Table 4:** The comparison of the quality of x-fit from x- and *v*-spaces respectively using squared sum of errors (SSE).

	SSE with Noisy Data		SSE with True Data	
$\sigma_{e}$	**x-Fit (U)**	**x-Fit (V)**	**x-Fit (U)**	**x-Fit (V)**
0.001	1.1743	0.6082	1.1753	0.6089
0.3	12.4677	12.6049	2.1946	0.8541
0.7	62.4582	63.4059	4.1855	0.8176
1.0	121.0864	120.8161	13.3478	1.1253
1.3	163.1339	264.3176	18.9582	3.1651

## Appendix A

In this appendix, we present some details on the integration of the exponential cubic spline. The integration is performed in two cases. In one case, one integrates the spline function between two knots, and it is referred to as $I_{\mathrm{full}}$. In the other case, one integrates the spline function from a knot to a random point before the next knot, and it is referred to as $I_{\mathrm{partial}}$. The two types of integrals yield

(A1) $$I_{\mathrm{full}}=\int_{\xi_{v_{k}}}^{\xi_{v_{k+1}}}S_{v}\left(t,\;f_{v},\;\lambda_{v},\;\xi_{v},\;E_{v}\right)dt,$$

with $k=1,\ldots ,E_{v}-1$, and

(A2) $$I_{\mathrm{partial}}=\int_{\xi_{v_{k}}}^{t_{i}}S_{v}\left(t,\;f_{v},\;\lambda_{v},\;\xi_{v},\;E_{v}\right)dt$$

with $k=1,\ldots ,E_{v}-1$, i=1,…,n, and $\xi_{v_{k}}\leq t_{i}<\xi_{v_{k+1}}$, respectively. The solutions to these two cases presented in Equations (A1) and (A2) can be found analytically and result in the following expressions:

(A3) $$\begin{array}{cc} I_{\mathrm{partial}} & =\frac{f_{v_{i+1}}}{2h_{v_{i}}}{\left(t_{i}-\xi_{v_{i}}\right)}^{2}+\frac{f_{v_{i}}\left(t_{i}-\xi_{v_{i}}\right)}{2h_{v_{i}}}\left(2\xi_{v_{i+1}}-t_{i}-\xi_{v_{i}}\right)\\ \; & -\frac{M_{v_{i}}\left(t_{i}-\xi_{v_{i}}\right)}{2\lambda_{v_{i}}^{2}h_{v_{i}}}\left(2\xi_{v_{i+1}}-t_{i}-\xi_{v_{i}}\right)\\ \; & +\frac{M_{v_{i}}}{\lambda_{v_{i}}^{3}\sinh\left(\lambda_{v_{i}}h_{v_{i}}\right)}\left\{\cosh\left(\lambda_{v_{i}}h_{v_{i}}\right)-\cosh\left[\lambda_{v_{i}}\left(t_{i}-\xi_{v_{i+1}}\right)\right]\right\} \end{array}$$

(A4) $$\begin{array}{cc} \; & +\frac{M_{v_{i+1}}}{\lambda_{v_{i}}^{3}\sinh\left(\lambda_{v_{i}}h_{v_{i}}\right)}\left\{\cosh\left[\lambda_{v_{i}}\left(t_{i}-\xi_{v_{i}}\right)\right]-1\right\}-\frac{M_{v_{i+1}}}{2\lambda_{v_{i}}^{2}h_{v_{i}}}{\left(t_{i}-\xi_{v_{i}}\right)}^{2}, \end{array}$$

and

(A5) $$I_{\mathrm{full}}=\frac{f_{v_{i}}h_{v_{i}}}{2}+\frac{f_{v_{i+1}}h_{v_{i}}}{2}-\frac{M_{v_{i}}h_{v_{i}}}{2\lambda_{v_{i}}^{2}}-\frac{M_{v_{i+1}}h_{v_{i}}}{2\lambda_{v_{i}}^{2}}+\frac{\left(M_{v_{i+1}}+M_{v_{i}}\right)}{\lambda_{v_{i}}^{3}}\tanh\left(\frac{\lambda_{v_{i}}h_{v_{i}}}{2}\right).$$

## Appendix B

In this appendix, as it is in the framework of Markov chain Monte Carlo (MCMC) methods, we present some details on the Delayed Rejection Adaptive Metropolis (DRAM) algorithm used to compute posterior distributions in our work. DRAM is a hybrid algorithm in which the concepts of Delayed Rejection (DR) and Adaptive Metropolis (AM) are combined for enhancing the performance of Metropolis-Hastings (MH)-type MCMC algorithms [35]. In the basic MH algorithm, upon the rejection of the new candidate at a (*j* + 1)th state, the sample remains at the current point (*j*). However, when the Delayed Rejection is introduced, upon rejection, another new candidate z is drawn. Then, one finds out whether it can be accepted. The AM feature, instead, is a global adaptive strategy, which is combined with the partial local adaptation caused by the DR strategy. The idea of AM is to tune the proposal distribution at each time step depending on the past. In other words, the covariance matrix of the proposal distribution depends on the history of the sample chain (sequence of samples). Usually, the adaptation starts after an initial period of time (0, *t*).

The basic steps of the DRAM algorithm are given in Algorithm A1 with an arbitrary initial point $\Theta^{\left(0\right)}$. The acceptance probabilities at steps 6 and 15 are shown in Equations (A6a) and (A6b), respectively.

(A6a) $$\begin{array}{cc} \alpha_{1}\left(x,\;y\right) & =\left\{\begin{array}{cc} \min\left[\frac{\pi \left(y\right)q\left(y,\;x\right)}{\pi \left(x\right)q\left(x,\;y\right)},\;1\right]\; & \mathrm{if}\pi \left(x\right)q\left(x,y\right)>0,\\ 1\; & \mathrm{otherwise}. \end{array}\right. \end{array}$$

(A6b) $$\begin{array}{cc} \alpha_{2}\left(x,\;y,\;z\right) & =\left\{\begin{array}{cc} \; & \min\left[\frac{\pi \left(z\right)}{\pi \left(x\right)}\frac{q\left(z,\;y\right)}{q\left(x,\;y\right)}\frac{q\left(z,\;y,\;x\right)}{q\left(x,\;y,\;z\right)}\frac{\left[1-\alpha_{1}\left(z,\;y\right)\right]}{\left[1-\alpha_{1}\left(x,\;y\right)\right]},\;1\right]\\ \; & \mathrm{if}\pi \left(x\right)q\left(x,\;y\right)q\left(x,\;y,\;z\right)\left[1-\alpha_{1}\left(x,\;y\right)\right]>0,\\ 1 & \;\mathrm{otherwise}. \end{array}\right. \end{array}$$

where q(θ) represents the proposal distribution and $\Theta^{\left(j\right)}$ represents the set of parameters of the proposal distribution at *j*-th iteration.

**Algorithm A1** DRAM
1: **for** j=1:N**do** 2: Generate *y* from $q\left(\Theta^{\left(j\right)},.\right)$ and *u* from U(0,1) 3: **if** *y* is outside $\left(L,\;U\right)$ **then** 4: Reject *y* (level 1) 5: **else** 6: Calculate $\alpha_{1}\left(\Theta^{\left(j\right)},\;y\right)$ 7: **if** $u\leq \alpha_{1}\left(\Theta^{\left(j\right)},\;y\right)$ **then** 8: $\Theta^{\left(j+1\right)}=y$ 9: **else** 10: Reject *y* (level 2) {starts delayed rejection} 11: Generate new candidate *z* from $q\left(y,.\right)$ 12: **if** *z* is outside $\left(L,\;U\right)$ **then** 13: Reject *z* (level 3) 14: **else** 15: Calculate $\alpha_{2}\left(\Theta^{\left(j\right)},\;y,\;z\right)$ and *u* from U(0,1) 16: **if** $u\leq \alpha_{2}\left(\Theta^{\left(j\right)},\;y,\;z\right)$ **then** 17: $\Theta^{\left(j+1\right)}=z$ 18: **else** 19: Reject *z* (level 4) 20: $\Theta^{\left(j+1\right)}=\Theta^{\left(j\right)}$ 21: **end if** 22: **end if** 23: **end if** 24: **end if** 25: **end for** 26: Return the values $\left\{\Theta^{\left(0\right)},\;\Theta^{\left(1\right)},\;\Theta^{\left(2\right)},\ldots ,\Theta^{\left(N\right)}\right\}$.
